# UV radiation sensitivity of bacteriophage PhiX174 - A potential surrogate for SARS-CoV-2 in terms of radiation inactivation

**DOI:** 10.3934/microbiol.2023023

**Published:** 2023-05-05

**Authors:** Laura Weyersberg, Florian Sommerfeld, Petra Vatter, Martin Hessling

**Affiliations:** Ulm University of Applied Sciences, Department of Medical Engineering and Mechatronics, Biotech-Lab, Albert Einstein-Allee 55, D-89081 Ulm, Germany

**Keywords:** SARS-CoV-2 surrogate, PhiX174, ultraviolet radiation, radiation disinfection, UVA, UVB, UVC, Far-UVC

## Abstract

To minimize health risks, surrogates are often employed to reduce experiments with pathogenic microorganisms and the associated health risk. Due to structural similarities between the enveloped RNA-viruses SARS-CoV-2 and Phi6, the latter has been established as a nonpathogenic coronavirus surrogate for many applications. However, large discrepancies in the UV log-reduction doses between SARS-CoV-2 and Phi6 necessitate the search for a better surrogate for UV inactivation applications. A literature study provided the bacteriophage PhiX174 as a potentially more suitable nonpathogenic coronavirus surrogate candidate. In irradiation experiments, the sensitivity of PhiX174 was investigated upon exposure to UV radiation of wavelengths 222 nm (Far-UVC), 254 nm (UVC), 302 nm (broad-band UVB), 311 nm (narrow-band UVB) and 366 nm (UVA) using a plaque assay. The determined log-reduction doses for PhiX174 were 1.3 mJ/cm^2^ @ 222 nm, 5 mJ/cm^2^ @ 254 nm, 17.9 mJ/cm^2^ @ 302 nm, 625 mJ/cm^2^ @ 311 nm and 42.5 J/cm^2^ @ 366 nm. The comparison of these results with published log-reduction doses of SARS-CoV-2 in the same spectral region, led to the conclusion that the bacteriophage PhiX174 exhibits larger log-reduction doses than SARS-CoV-2, nevertheless, it is a better UV-surrogate at 222 nm (Far-UVC), 254 nm (UVC) and 302 nm (UVB) than the often applied Phi6.

## 1. Introduction

As of March 15, 2023, approximately 670,000,000 people worldwide had been infected with SARS-CoV-2, and nearly 6.8 million people have already died from COVID-19 [1]. Even the development of promising vaccines, high vaccination rates and international and national pandemic measures like the German Infectious Diseases Protection Act [2], could not stop the spread of infection. Most recently, mutations and subtypes such as Omicron ensured milder courses but also an even more rapid spread. The pandemic has again created a special sensitivity to the emergence of diseases of zoonotic origin. Since 2020, a steadily increasing number of cases of a subtype of zoonotic avian influenza HPAIV has again been reported, fortunately affecting only a few hundred people worldwide to date [3]. In May 2022, an outbreak of MPXV, better known as monkeypox, caused “a public health emergency of international proportions” [4].

The greatest concern remains a potential overload of the health care system as was seen repeatedly during the SARS-CoV-2 pandemic. Various pandemic containment measures and implementation of hygiene concepts were recommended. For the elimination of microorganisms and viruses, radiation disinfection has been established as a successful method [5],[6]. Ultraviolet (UV) radiation is divided into the wavelength-specific ranges of UVA (315–400 nm) , UVB (280–315 nm) and UVC (200–280 nm) [7],[8]. Especially interesting for applications in the field of medical technology is a subtype of UVC radiation, which is called Far-UVC. It is defined by a wavelength between 200–230 nm and known to be less harmful to human skin and eyes [9]–[15].

However, the ability to conduct SARS-CoV-2 irradiation experiments, e.g., for the development and test of new air disinfection systems is limited as biosafety level 3 facilities are required, which are only available to a limited extent. Comparative data reveals that irradiation of other coronaviruses, such as human coronavirus HCoV-OC43 or mouse hepatitis virus MHV, yields similar results, making them highly suitable surrogates [16]. However, work with these coronaviruses is also avoided in biosafety laboratories, as accidents have already occurred in the past [17],[18]. The application of an appropriate surrogate encourages the development of radiation disinfection devices such as surface and air disinfection systems by reducing capital costs, as manufacturers often have limited access to biosafety level 2 or 3 laboratories. So even less harmful but still pathogenic coronaviruses are not desired.

Therefore, the identification of an application-specific; nonpathogenic SARS-CoV-2 surrogate for radiation disinfection research and development is of great importance. By facilitating the evaluation and approval of prototypes through verification with surrogates, development potentials can be exploited. This contributes significantly to containing the spread of pathogens and supporting the healthcare system.

Previously, the bacteriophage Phi6 was considered a suitable coronavirus surrogate for irradiation experiments, as both are enveloped RNA viruses and Phi6 has been applied as a coronavirus surrogate in many applications [19]–[28]. However, Phi6 proved to be much less UV sensitive compared to SARS-CoV-2 and is therefore no suitable coronavirus surrogate for UV irradiation / disinfection experiments [29].

In a preceding literature study, we have already determined median UV log-reduction doses for SARS-CoV-2 in liquids [29]. For the UVC-range from 251–270 nm around the RNA absorption peak at approximately 260 nm, the median SARS-CoV-2 log-reduction dose was 1.7 mJ/cm^2^. For Far-UVC, UVB and UVA the determined median SARS-CoV-2 log-reduction doses were 1.15 mJ/cm^2^ (@ 222 nm), 10.7 mJ/cm^2^ (@ 297–308 nm) and 2569 mJ/cm^2^ (@ 365 nm / 366 nm), respectively [29].

Our selection of an alternative virus as SARS-CoV-2 surrogate candidate for disinfection experiments is based on published UVC log-reduction doses as well as apathogenicity, which allows work in most microbiological laboratories. A promising surrogate candidate should also have a median UVC log-reduction dose of about 1.7 mJ/cm^2^ - or slightly higher as a safety margin.The choice was limited to known phages besides Phi6 like MS2, PhiX174, Qβ, T1, T2, T3, T4 and T7 and a few more, for which at least some UVC data was published. For PhiX174 UVC irradiation experiments with irradiation wavelengths of around 260 nm, several different authors determined inactivation results with log-reduction doses around 2.4 mJ/cm^2^ (median; the single values are listed in Table 1), which is quite near the SARS-CoV-2 log-reduction dose of 1.7 mJ/cm^2^ in the same spectral region. For all other phages, the retrieved log-reduction doses were further away and/or the data was considered less reliable because there were less publications.

Therefore, in this study, Far-UVC (222 nm), UVC (254 nm), UVB (302/311 nm) and UVA (366 nm) log-reduction doses for phage PhiX174 were determined experimentally and compared to the above mentioned literature values for SARS-CoV-2 to evaluate its suitability as a surrogate in UV disinfection applications.

## 2. Materials and methods

The bacteriophage PhiX174 (DSM 4497) and the recommended host *Escherichia coli* (DSM 13127) were obtained from DSMZ (German Collection of Microorganisms and Cell Cultures, Braunschweig, Germany).

A bacterial colony of *E. coli* was cultured in 3 mL Luria Bertani (LB) medium for 3 hours at 37 °C and 170 rpm. Thereafter, the culture exhibited an optical density of 0.15 at 600 nm, which corresponded to 1 × 10^9^ colony forming units (CFU)/mL.

**Figure 1.**
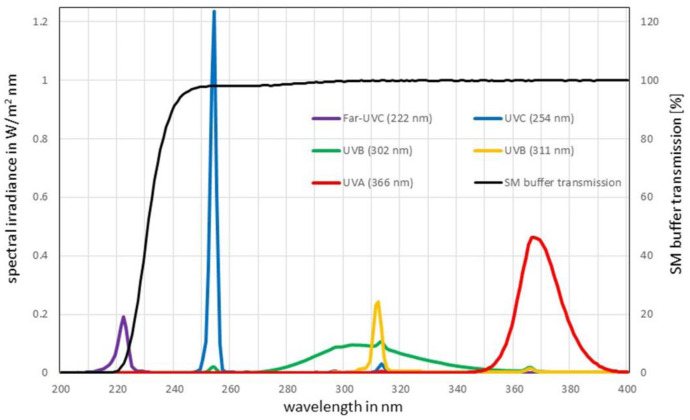
Left y-axis: spectral irradiance of the applied UV sources at defined irradiation distances with emission peaks at 222 nm (Far-UVC), 254 nm (UVC), 302 nm (broad-band UVB), 311 nm (narrow-band UVB) and 366 nm (UVA); right y-axis: absorption spectrum of SM buffer for an optical path length of 10 mm. The transmittances relevant for this work were all >98%, with the exception of 222 nm where it was only 3.2% for this path length. (The path length during the 222 nm irradiation experiment was only 1 mm).

A bacteriophage stock solution with a titer of 10^8^ viruses/mL in saline magnesium (SM) buffer was prepared as described by Sambrook and Russel [30]. The transmittance of the SM buffer was measured using a Specord Plus absorption spectrometer of Analytik Jena (Jena, Germany) with distilled water in a 10 mm long quartz cuvette as reference. As illustrated in Figure 1, significant absorption was only recorded in the Far-UVC range below 230 nm.

A “*UV222™*” lamp of UV Medico (Aarhus, Denmark) was applied for 222 nm Far-UVC irradiation. The radiation source for the UV wavelengths 254 nm, 302 nm and 366 nm was the “*3UV-36 lamp*” of Analytik Jena. As addition to the broad-band 302 nm UVB lamp (spectral width 40 nm), a narrow-band UVB source type “*UVB medical PLS*” from Osram (Munich, Germany) with a peak wavelength at 311 nm (spectral width 4 nm), was employed. For defined distances, the spectral irradiances, given in Figure 1, were measured using the “*CAS140D*” photometer of Instrument Systems (Munich, Germany).

As illustrated by Figure 1, there are noticeable discrepancies in the emission spectra between both UVB radiation sources. The 302 nm lamp emits a broad spectrum with the main emission in the UVB range between 280 and 315 nm, but with some UVC and UVA contributions. In contrast to this, the 311 nm narrow-band UVB lamp actually exhibits only UVB radiation. The measured UV irradiances at these wavelengths were 0.098 mW/cm^2^ (Far-UVC), 0.366 mW/cm^2^ (UVC), 0.429 mW/cm^2^ (broad-band UVB), 0.192 mW/cm^2^ (narrow-band UVB) and 1.797 mW/cm^2^ (UVA).

For the irradiation experiments, the phage stock solution was diluted to a concentration of 10^7^ plaque forming units per mL (PFU/mL) in SM buffer before each experiment. A sample of 3 mL was filled into quartz beakers with a diameter of 22 mm for each irradiation at 254 nm, 302 nm, 311 nm and 366 nm. To account for the strong absorption of the SM buffer at 222 nm, the sample volume had to be reduced to a 1 mm fill level to ensure an average transmission of approximately 84%. Therefore, the irradiation at 222 nm was performed in a quartz Petri dish with a diameter of 27 mm to provide sufficient solution for sampling, which would have been too small at a height of 1 mm in a quartz beaker.

The temperature of the samples was kept constant throughout the experiments. During the long time UVA radiation at 366 nm, cooling by a 20 °C water bath (Thermocell of Biozyme Scientific, Hessisch Oldendorf, Germany) was necessary to prevent sample heating. All other experiments were performed at room temperature due to their short duration.

After fixed periods of time, 100 µL samples of the irradiated phage suspension were taken and the irradiation was then continued. Three samples were taken at each of the fixed times and three independent experimental runs were performed.

Irradiated and non-irradiated phage solutions were diluted up to 10^–6^ in each of three dilution series (900 µL SM + 100 µL sample). 100 µL of each phage sample was mixed with 100 µL bacteria suspension, which was previously further diluted 1:10 with phosphate buffered saline. After an incubation time of 10 min, the phage-bacteria-suspension was mixed with soft agar and poured on to LB medium agar plates based on the “double agar layer technique” referred to by the DSMZ [31],[32]. Soft agar was based on LB medium and contained only 6 g/L of agar, which lead to a low viscosity. The soft agar had a temperature of 50 °C so that the consistency of the agar was appropriately liquid for the casting process, but the bacteria and phages did not get denatured. The incubation of the agar plates took place in an incubator at a temperature of 37 °C for a period of 4–6 hours.

After the incubation, the lysed plaques in the bacterial lawn were photographed and counted. Comparison of the PFU after irradiation with the initial phage concentration indicated the achieved reductions.

## 3. Results

### 3.1. 222 nm irradiation (Far-UVC)

**Figure 2.**
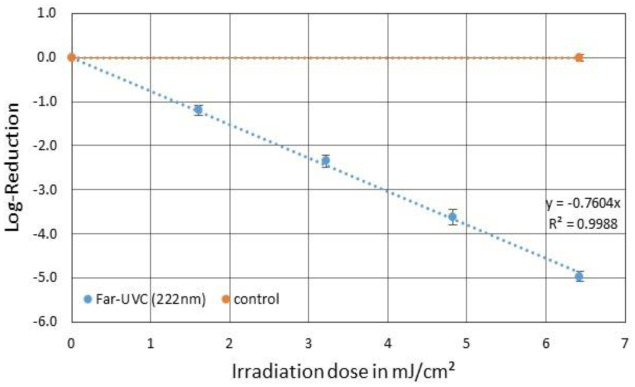
Averaged reduction of PhiX174 in SM buffer by 222 nm Far-UVC irradiation (blue) and the corresponding unirradiated control (orange). The error bars indicate the standard deviation of the triplicate. The average Far-UVC irradiation dose for a one log-reduction was 1.3 mJ/cm^2^.

For a total irradiation Far-UVC dose of ~6.5 mJ/cm^2^, which took 80 s, a PhiX174 reduction of 4.97 log levels was observed as illustrated in Figure 2. This resulted in an average 222 nm PhiX174 log-reduction dose of 1.3 mJ/cm^2^ and the inactivation rate constant was 0.76 cm^2^/mJ.

### 3.2. 254 nm irradiation (UVC)

**Figure 3.**
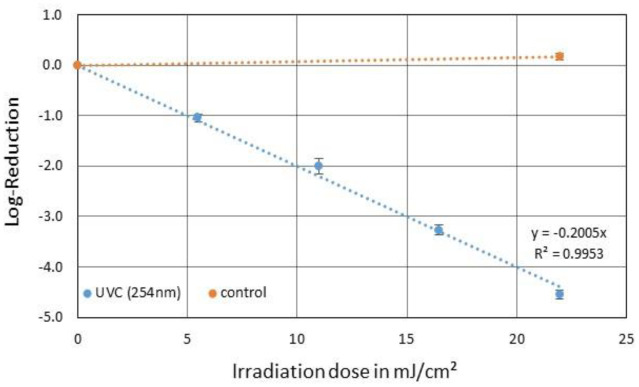
Averaged reduction of PhiX174 in SM buffer by irradiation with 254 nm UVC (blue) and the corresponding control (orange). The error bars indicate the standard deviation of the triplicate. The average irradiation dose for a one log-reduction was 5.0 mJ/cm^2^.

With an irradiation time of 60 s and a resulting UVC irradiation dose of ~22 mJ/cm^2^, a reduction of 4.54 log levels was detected for PhiX174. The resulting average UVC log-reduction dose was therefore 5.0 mJ/cm^2^ and the inactivation rate constant was 0.20 cm^2^/mJ (Figure 3).

### 3.3. 302 nm irradiation (broad-band UVB)

**Figure 4.**
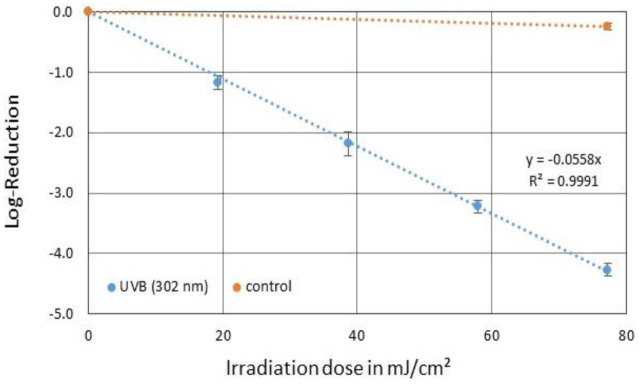
Averaged reduction of phage PhiX174 in SM buffer by irradiation with a broad-band 302 nm UVB source (blue) and the corresponding control (orange). The error bars indicate the standard deviation of the triplicate. The average irradiation dose for a one log-reduction was 17.9 mJ/cm^2^.

180 seconds and the resulting irradiation dose of ~77 mJ/cm^2^, a reduction of 4.27 log levels could be determined for PhiX174 (Figure 4). The resulting average log-reduction dose was therefore 17.9 mJ/cm^2^ and the inactivation rate constant was 0.056 cm^2^/mJ.

### 3.4. 311 nm irradiation (narrow-band UVB)

**Figure 5.**
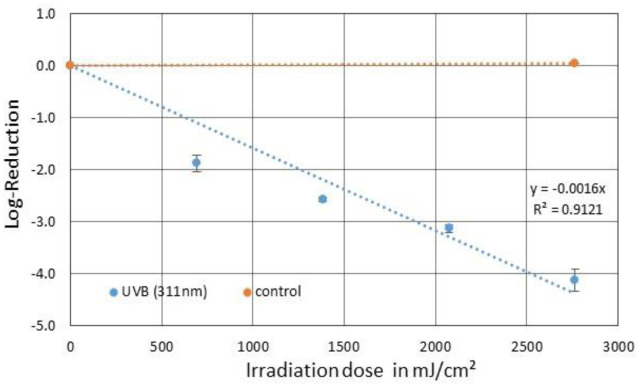
Averaged reduction of PhiX174 in SM buffer by irradiation with a narrow-band 311 nm UVB source (blue) and the corresponding control (orange). The error bars indicate the standard deviation of the triplicate. The average irradiation dose for a one log-reduction was 625 mJ/cm^2^.

After an irradiation time of 4 hours and a total irradiation dose of 2765 mJ/cm^2^, a reduction of 4.12 log levels was observed for PhiX174, which can be seen in Figure 5. The resulting average log-reduction dose was 625 mJ/cm^2^ and the inactivation rate constant was 0.0016 cm^2^/mJ.

### 3.5. 366 nm irradiation (UVA)

**Figure 6.**
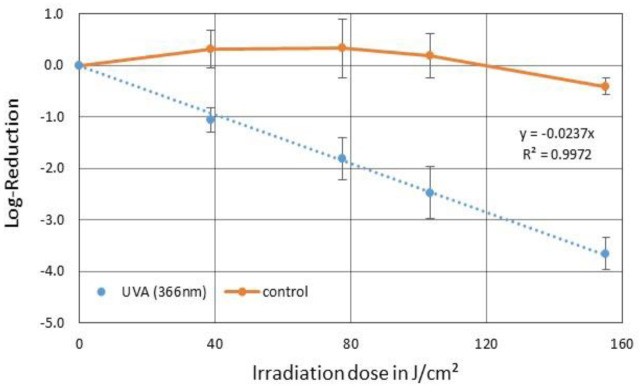
Averaged reduction of PhiX174 in SM buffer by irradiation with 366 nm UVA (blue) and the corresponding control (orange). The error bars indicate the standard deviation of the triplicate. The average irradiation dose for one log-reduction was 42.5 J/cm^2^.

With an irradiation time of 24 hours and a total irradiation dose of ~155 J/cm^2^, a PhiX174 reduction of 3.65 log levels was detected (Figure 6). This resulted in an average irradiation dose of 42.5 J/cm^2^, which was necessary to reduce the phages by one log level. The inactivation rate constant was 0.024 cm^2^/J.

## 4. Discussion

**Figure 7.**
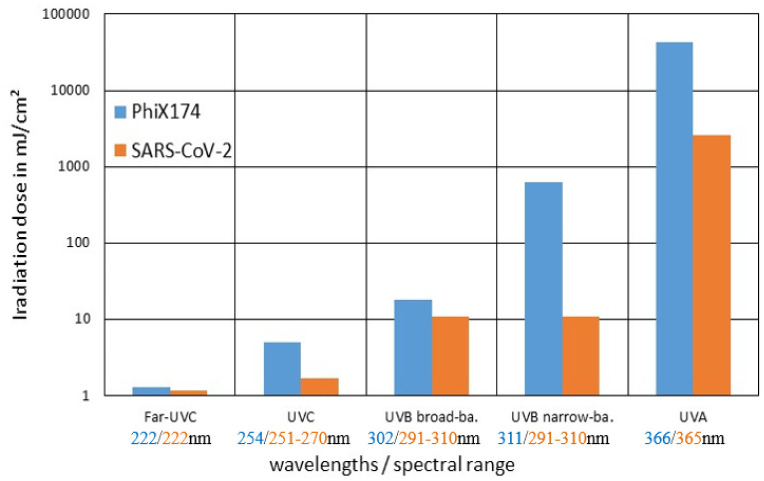
Logarithmically scaled comparison between measured PhiX174 (blue, this investigation) and SARS-CoV-2 (orange, literature values from [29]) log-reductions doses for the different spectral ranges / wavelengths.

In all PhiX174 UV irradiation experiments, a more or less exponential inactivation was observed. The log-reduction doses determined from these are illustrated in Figure 7 and in Table 1, together with retrieved PhiX174 literature values and the associated SARS-CoV-2 log-reduction doses taken from [29].

Our PhiX174 results are mostly in good agreement with the literature values. However in the important UVC range, the here determined log-reduction dose of 5 mJ/cm^2^ is twice as high as the median of published values of 2.4 mJ/cm^2^. Both values are higher than the previously determined median SARS-CoV-2 log-reduction dose of 1.7 mJ/cm^2^. However, both values are much closer to the SARS-CoV-2 log-reduction dose than Phi6 with its corresponding median UVC log-reduction dose of 31.5 mJ/cm^2^ [29].

In the Far-UVC range, there is not much published PhiX174 data, but the here determined PhiX174 log-reduction dose is in agreement with the literature and fits very well to the associated SARS-CoV-2 value.

The peak emission wavelengths of the two employed UVB sources differ by only 9 nm and therefore one would have expected similar results for the irradiation experiments. However, the PhiX174 inactivation by the broad-band 302 nm lamp, is about 35 times higher than for the narrow-band 311 nm lamp. The reason for this effect might be the UVC emission parts of the broad-band 302 nm lamp. The PhiX174 log-reduction dose of 17.9 mJ/cm^2^ obtained with the 302 nm lamp is within the literature values, and in the same order of magnitude as the corresponding SARS-CoV-2 log-reduction dose of 10.7 mJ/cm^2^ [29].

No UVA data was found in the literature on the irradiation of PhiX174. The UVA log-reduction dose of 42.5 J/cm^2^ determined in the present work is significantly higher than the associated SARS-CoV-2 value of 2.57 J/cm^2^ [29]. Therefore, PhiX174 seems to be no suitable SARS-CoV-2 UVA surrogate.

**Table 1.** Median UV log-reduction doses for PhiX174 in liquid medium for different wavelengths with corresponding SARS-CoV-2 medians in brackets for comparison. The SARS-CoV-2 log-reduction doses were taken from [29].

PhiX174 median log-reduction dose in mJ/cm^2^ [spectral range]	wavelength in nm	single log-reduction dose in mJ/cm^2^	medium
Far-UVC [230 nm]: PhiX174: 2.2 (SARS-CoV-2: 1.15)	*222*	1.3*	SM buffer (this study)*
	230	2.2	PBS [33]
UVC [254–265 nm]: PhiX174: 2.4 (SARS-CoV-2: 1.7)	254	1.5	butanol water mix [34]
	254	1.8	KCl [35]
	254	2.0	unknown [36]
	254	2.2	water [37]
	254	2.3	PBS [38]
	254	2.3	NaCl [39]
	254	2.5	sterile water [40]
	254	2.5	waste water [41]
	254	3.0	waste water [42]
	254	3.0	PBS [43]
	254	3.3	PBS [44]
	*254*	5.0*	SM buffer (this study)*
	254	10.2	PBS [45]
	265	1.4	PBS [33]
UVB [280–301 nm]: PhiX174: 18.1 (SARS-CoV-2: 10.7)	280	2.1**	KCl [35]
	289	4.9	PBS [33]
	301	31.3	KCl [35]
	*302 (broad-ba.)*	17.9*	SM buffer (this study)*
	*311 (narrow-ba.)*	625*	SM buffer (this study)*
UVA [366 nm]: PhiX174: - (SARS-CoV-2: 2569)	*366*	42500*	SM buffer (this study)*

*This study; not included in the median determination; **between UVC and UVB and therefore not included in the median determination for UVC or UVB

As a limitation to our general statements on log-reduction doses in the UV spectral ranges, Far-UVC, UVC, UVB and UVA, we would like to point out that for each of these spectral ranges we applied only one or two typically implemented wavelengths or radiation sources. The photoinactivation properties of SARS-CoV-2 and PhiX174 will not be constant within these UV spectral regions but change with wavelength. However, the log-reduction doses reported here should provide a reasonable reference point for other wavelengths in these UV ranges.

## 5. Conclusions

For Far-UVC (222 nm), UVC (254 nm) and UVB (302 nm), the PhiX174 log-reduction doses are similar or slightly higher than the corresponding SARS-CoV-2 doses. This makes PhiX174 a much better SARS-CoV-2 surrogate in Far-UVC, UVC or UVB irradiation experiments than the usually applied coronavirus surrogate Phi6.

However, based on the limited available data, the same cannot be claimed for the UVA around 366 nm. A good SARS-CoV-2 UVA surrogate still has to be found.

## References

[1] (2023). Center for Systems Science and Engineering at Johns Hopkins University (Johns Hopkins University & Medicine Coronavirus Resource Center), COVID-19 Dashboard.

[2] Gerhardt J (2022). Infektionsschutzgesetz (IfSG): Kommentar.

[3] (2023). Robert Koch Institut, Zoonotischen Influenza bei Menschen.

[4] (2023). Robert Koch Institut, Internationaler Mpox-Ausbruch: Einschätzung der Situation in Deutschland.

[5] Singh D, Soorneedi AR, Vaze N (2023). Assessment of SARS-CoV-2 surrogate inactivation on surfaces and in air using UV and blue light-based intervention technologies. J Air Waste Manag Assoc.

[6] Heßling M, Hönes K, Vatter P (2020). Ultraviolet irradiation doses for coronavirus inactivation - review and analysis of coronavirus photoinactivation studies. GMS Hyg Infect Control.

[7] Seyer A, Sanlidag T (2020). Solar ultraviolet radiation sensitivity of SARS-CoV-2. Lancet Microbe.

[8] Kowalski W (2009). Ultraviolet germicidal irradiation handbook: UVGI for air and surface disinfection.

[9] Buonanno M, Welch D, Shuryak I (2020). Far-UVC light (222 nm) efficiently and safely inactivates airborne human coronaviruses. Sci Rep.

[10] Zwicker P, Schleusener J, Lohan SB (2022). Application of 233 nm far-UVC LEDs for eradication of MRSA and MSSA and risk assessment on skin models. Sci Rep.

[11] Buonanno M, Welch D, Brenner DJ (2021). Exposure of human skin models to KrCl excimer lamps: the impact of optical filtering. Photochem Photobiol.

[12] Welch D, Aquino de Muro M, Buonanno M (2022). Wavelength-dependent DNA photodamage in a 3-D human skin model over the far-UVC and germicidal UVC wavelength ranges from 215 to 255 nm. Photochem Photobiol.

[13] Welch D, Kleiman NJ, Arden PC (2023). No evidence of induced skin cancer or other skin abnormalities after long-term (66 week) chronic exposure to 222-nm far-UVC radiation. Photochem Photobiol.

[14] Eadie E, Barnard IMR, Ibbotson SH (2021). Extreme exposure to filtered far-UVC: a case study. Photochem Photobiol.

[15] Hickerson RP, Conneely MJ, Hirata Tsutsumi SK (2021). Minimal, superficial DNA damage in human skin from filtered far-ultraviolet C. Br J Dermatol.

[16] Boegel SJ, Gabriel M, Sasges M (2021). Robust evaluation of ultraviolet-C sensitivity for SARS-CoV-2 and surrogate coronaviruses. Microbiol Spectr.

[17] Normile D (2004). Mounting lab accidents raise SARS fears. Science.

[18] Della-Porta T (2008). Laboratory accidents and breaches in biosafety – they do occur!. Microbiol Aust.

[19] Cadnum JL, Li DF, Jones LD (2020). Evaluation of ultraviolet-C light for rapid decontamination of airport security bins in the era of SARS-CoV-2. Pathog Immun.

[20] Prussin AJ, Schwake DO, Lin K (2018). Survival of the enveloped virus Phi6 in droplets as a function of relative humidity, absolute humidity, and temperature. Appl Environ Microbiol.

[21] Casanova LM, Weaver SR (2015). Evaluation of eluents for the recovery of an enveloped virus from hands by whole-hand sampling. J Appl Microbiol.

[22] Ye Y, Ellenberg RM, Graham KE (2016). Survivability, partitioning, and recovery of enveloped viruses in untreated municipal wastewater. Environ Sci Technol.

[23] Ma B, Gundy PM, Gerba CP (2021). UV inactivation of SARS-CoV-2 across the UVC spectrum: KrCl* excimer, mercury-vapor, and light-emitting-diode (LED) sources. Appl Environ Microbiol.

[24] Silverman AI, Boehm AB (2020). Systematic review and meta-analysis of the persistence and disinfection of human coronaviruses and their viral surrogates in water and wastewater. Environ Sci Technol Lett.

[25] Aquino de Carvalho N, Stachler EN, Cimabue N (2017). Evaluation of Phi6 persistence and suitability as an enveloped virus surrogate. Environ Sci Technol.

[26] Whitworth C, Mu Y, Houston H (2020). Persistence of bacteriophage phi 6 on porous and nonporous surfaces and the potential for its use as an Ebola virus or coronavirus surrogate. Appl Environ Microbiol.

[27] Lytle CD, Budacz AP, Keville E (1991). Differential inactivation of surrogate viruses with merocyanine 540. Photochem Photobiol.

[28] Costa L, Faustino MAF, Neves MGPMS (2012). Photodynamic inactivation of mammalian viruses and bacteriophages. Viruses.

[29] Weyersberg L, Klemens E, Buehler J (2022). UVC, UVB and UVA susceptibility of Phi6 and its suitability as a SARS-CoV-2 surrogate. AIMS Microbiol.

[30] Sambrook J, Russell DW (2001). Molecular Cloning: A Laboratory Manual. Q Rev Biol.

[31] Cormier J, Janes M (2014). A double layer plaque assay using spread plate technique for enumeration of bacteriophage MS2. J Virol Methods.

[32] Vatter P, Hoenes K, Hessling M (2021). Blue light inactivation of the enveloped RNA virus Phi6. BMC Res Notes.

[33] Setlow R, Boyce R (1960). The ultraviolet light inactivation of ΦX174 bacteriophage at different wave lengths and pH's. Biophys J.

[34] David CN (1964). UV inactivation and thymine dimerization in bacteriophage phi x. Z Vererbungsl.

[35] Giese N, Darby J (2000). Sensitivity of microorganisms to different wavelengths of UV light: implications on modeling of medium pressure UV systems. Water Res.

[36] Yarus M, Sinsheimer RL (1964). The UV-resistance of double-stranded PhiX174 DNA. J Mol Biol.

[37] Rodriguez RA, Bounty S, Beck S (2014). Photoreactivation of bacteriophages after UV disinfection: role of genome structure and impacts of UV source. Water Res.

[38] Battigelli DA, Sobsey MD, Lobe DC (1993). The inactivation of hepatitis a virus and other model viruses by UV irradiation. Water Sci Technol.

[39] Sommer R, Haider T, Cabaj A (1998). Time dose reciprocity in UV disinfection of water. Water Sci Technol.

[40] Zuo X, Chu X, Hu J (2015). Effects of water matrix on virus inactivation using common virucidal techniques for condensate urine disinfection. Chemosphere.

[41] Sommer R (2001). Inactivation of bacteriophages in water by means of non-ionizing (uv-253.7nm) and ionizing (gamma) radiation: a comparative approach. Water Res.

[42] Ho J, Seidel M, Niessner R (2016). Long amplicon (LA)-qPCR for the discrimination of infectious and noninfectious phix174 bacteriophages after UV inactivation. Water Res.

[43] Proctor WR, Cook JS, Tennant RW (1972). Ultraviolet photobiology of Kilham rat virus and the absolute ultraviolet photosensitivities of other animal viruses: influence of DNA strandedness, molecular weight, and host-cell repair. Virology.

[44] Lee HS, Sobsey MD (2011). Survival of prototype strains of somatic coliphage families in environmental waters and when exposed to UV low-pressure monochromatic radiation or heat. Water Res.

[45] Nuanualsuwan S, Mariam T, Himathongkham S (2002). Ultraviolet inactivation of feline calicivirus, human enteric viruses and coliphages. Photochem Photobiol.

